# History and Perspectives of AOFNMB

**DOI:** 10.7508/aojnmb.2013.01.002

**Published:** 2013

**Authors:** Henry Hee-Seung Bom

**Affiliations:** President of AOFNMB

In response to the historic document known as the Pisa Declaration in 1967, the Asia and Oceania Federation of Nuclear Medicine and Biology (AOFNMB) was inaugurated provisionally in October 1969 in Tokyo at a gathering of 21 delegations from the nine member countries and regions. The Second assembly of the AOFNMB was held in Tokyo in 1974 during the first World Congress of Nuclear Medicine and Biology (WCNMB), while Sydney was decided to be the hosting city of the first congress. The first Asia and Oceania Congress of Nuclear Medicine and Biology (AOCNMB) was held successfully in Sydney in September 1976. Prof. IPC (Provan) Murray was the Convenor of the meeting and Dr. Andrew McLaughlin was the Treasurer. There were 200 attendees at the meeting. The Congress was opened by ringing the congress bell once, which has become a tradition as a symbol of continuity. The congress bell was donated by the Japanese Society of Nuclear Medicine (JSNM) and every congress is opened by ringing the bell the number of times according to the order of the AOCNMB.

The second congress was held at the Philippine International Convention Center in Manila, Philippines in November 1980. The organizing committee included Dr. Leland Villadolid as the president, Dr. Virgilio Gonzales as the vice chairman, Dr. Flora Pascasio as the secretary general, and Dr. Celia Talusan as the treasurer.

The third congress was held in Seoul, Korea in August 27-31, 1984. It was the first international congress of medicine in Korea led by Prof. Munho Lee as the president, Prof. Chang-Soon Koh as the secretary general, and Prof. Myung-Chul Lee as the secretary. Approximately 700 attendants from 25 countries attended the congress. A workshop of International Atomic Energy Agency (IAEA) on the quality assurance of nuclear medicine instruments was followed as a post-congress program at the Seoul National University Hospital.

The fourth congress was held in Taipei, Taiwan in November 1-4, 1988. Prof. Peter Shin-Hwa Yeh was the president, Prof. Si-Jung Yeh was the vice president and Dr. Ren-Shyan Liu was the secretary general. A pre-congress educational program was organized by Prof. Samuel D. J. Yeh.

The fifth congress was held in Jakarta, Indonesia in 1992. Attendants were invited to the Presidential Palace of the Republic of Indonesia to meet President Soeharto and moved to Bali island for post-congress meeting. The congress was co-chaired by Prof. Johan S. Masjhur of Indonesia and Prof Abdul Dayem of Kuwait.

The sixth congress was held at the Kyoto International Convention Center, Kyoto, Japan in October 1-5, 1996. The president and congress chairman was Prof. Kanji Torizuka, and the secretary general was Prof. Junji Konishi. It was a joint meeting with the 36^th^ Annual Meeting of Japanese Society of Nuclear Medicine (JSNM). It is remembered by the largest attendants, where 1500 people attended from 41 countries. Dr. Gopinathan G. Nair, head of the Nuclear Medicine Section at the International Atomic Energy Agency - a co-sponsor of the congress - mentioned that he identified three fronts on which progress will need to be made in marching forward to the next century, namely, reducing the cost of nuclear medicine products and services, sustaining the quality to make it more competitive, demonstrating appropriateness, and emerging as a complementary and competing modality of investigation.

The seventh congress in conjunction with the 4th International Congress of Nuclear Oncology was held in Istanbul, Turkey under the auspices of the 9th Turkish Republic President his Excellency Suleyman Demirel on October 1-5, 2000. ICEC Lütfi Kirdar Convention and Exhibition Center housed around 700 participants. The president of the congress was Prof. Dr. Coskun Bekdik and the scientific committee was chaired by Prof. Dr. Ali Tan Isitman.

The eighth congress was held in Beijing, China in October 9-13, 2004. Around 500 attendants from 30 countries attended the congress. Prof. Xiu-Jie Liu was the president and Prof. Zuo-Xiang He was the secretary general. Post-congress satellite meetings were held in Shanghai and Hong Kong in October 15-17.

The ninth congress was held at the Ashok hotel in New Delhi, India with the theme of ‘globalization of nuclear medicine’ from October 31^st^ to November 4^th^, 2008. The president was Prof. Arun Malhotra from All India Institute of Medical Sciences. Delegates from 38 countries attended the meeting with 789 participants; it was inaugurated by the world renowned missile scientist and the President of India, Dr. Abdul J Kalam. Twelve CME and 30 scientific sessions comprising of oral and e-poster presentations, highlighting the development of Nuclear Medicine in Asia Oceania and globe, were presented during the congress.

The tenth congress was held in Tehran, Iran in May 16-20, 2012. Organizers were President Dr. Mohsen Saghari, Vice President and Chair of the scientific committee Dr. Mohammad Eftekhari, and Treasurer Dr. Seyed Rasoul Zakavi. The theme was ‘Nuclear Medicine All Over The World’. The total number of participants was 1027 from 36 countries, and 382 papers were presented [1].

I was elected as the 11^th^ President of the Federation at the 10^th^ AOFNMB Governing Council meeting which was held during the 10^th^ Congress. The interval of the congresses was decided to shorten to 2 years beginning from 2015: the 11^th^ Congress will be held in Jeju, Korea in 2015 and the 12^th^ Congress in Yokohama, Japan in 2017. Further changes were made at the National Delegate Assembly which was held during the Annual General Meeting of the Asian Regional Cooperative Council for Nuclear Medicine (ARCCNM) in October 25, 2012 in Seoul, Korea. The Charter was amended to have individual membership, to shorten the interval of the Congresses to two years by 2023 and to one year thereafter, to separate the jobs of the President and Congress Chairman, to publish an official journal with the name of the Asia Oceania Journal of Nuclear Medicine and Biology (AOJNMB), and to locate a permanent office in Korea.

In reality, AOFNMB is confronted with serious challenges to overcome. As I have discussed elsewhere, heterogeneity of nuclear medicine practice is one of the biggest challenges in Asia [2]. The activity of nuclear medicine practice is not exactly correlated to the size of the economy of the country. Public awareness, man power, as well as leadership are needed to promote nuclear medicine in each country. The role of AOFNMB to reduce heterogeneity of nuclear medicine practice and research has been limited. A congress every four years was not enough to communicate and exchange information among the member states. Thirty individuals sought other chances such as congresses of the Society of Nuclear Medicine (SNM) and European Association of Nuclear Medicine (EANM) to communicate and obtain information. Unfortunately, many Asian scientists and young doctors stay in their countries because they cannot afford frequent travels to United States or European countries. More frequent academic and educational meetings in Asia or Oceania are definitely needed. This is the reason for the organization of annual meetings in this region in the near future.

There are several regional meetings in Asia Oceania: Annual General Meeting of the Asia Regional Cooperative Council for Nuclear Medicine (ARCCNM), biennial meeting of the Far East Asian countries named by the CJK congress, Australia-New Zealand society meeting, and Gulf regional meeting. IAEA supported many regional training courses of nuclear medicine which served as a key engine to start nuclear medicine in many developing countries. Regional Cooperative Agreement (RCA) among Asian countries also provided projects of education and training for nuclear medicine related personnel. For example, RCA and United Nations Development Program (UNDP) collaboratively operate a training course on ‘Promoting and Accelerating Nuclear SPECT/PET Imaging Technologies in the Region’ from 2011 to 2013. There are some institutional efforts to share knowledge and experience in nuclear medicine. One good example is the Nuclear Medicine Update program of the Singapore General Hospital organized by Prof. Ajit Padhy every year.

Problems of the meetings and courses include language and finance. The meetings and courses are held only in English which can be a barrier to many Asian people. Translation of teaching materials is still not active in most countries. Financial support is limited to a few individuals. ARCCNM supports 25 young investigators each time. IAEA and RCA support less than 20 people for each course. Global economic crisis makes financial support even more difficult especially from the business side. Many companies reduce their budgets to support academic activities. Webinar is an attractive alternative to reduce the cost but maintain educational activities. IAEA, SNM, EANM, and AOFNMB are working to activate education through webinars.

Coordination between each training course is also a challenge. ARCCNM started the Asian School of Nuclear Medicine (ASNM) as an educational arm in 2003. The goal of ASNM is to facilitate the spread of knowledge of nuclear medicine in Asia by fostering nuclear medicine education in Asian countries through promotion of training for nuclear medicine physicians, technologists, radiopharmacists, medical physicists, and other allied professionals. ASNM cooperates with government agencies, universities, national societies, and industry partners. ASNM is assisting in national and regional training courses, awarding continuing professional education (CPE) points, providing regional experts for advanced educational programs, standardizing nuclear medicine education, training throughout the ARCCNM member states, and working towards awarding diplomas/degrees of continuing education (CE) units in association with recognized universities/hospitals by distance learning, practical attachments, and other scholarly activities. Since its establishment, ASNM has been actively involved in several teaching courses and activities. It has initiated some national training programs in nuclear medicine and has spearheaded educational sessions in various regional and national congresses. Furthermore, ASNM has initiated the survey of training programs in the member states, which will be critical in the development of a template for a standardized program for nuclear medicine in the region. Data collection on human resources (MD, PhD, Tech, etc.), equipment and instrumentation, procedures and training formats are likewise part of the effort to come up with a standardized training program. ASNM actively joins the training programs of the IAEA in Asia. [3] It is the responsibility of AOFNMB to broaden the activity of ASNM to other Asian regions including Middle and Central East.

Providing trainees with the knowledge and ability of conducting nuclear medicine applications according to global standards is needed to satisfy customers or the general population in Asian countries. The European Board of Nuclear Medicine (EBNM) is a good example of qualifying trainees. ARCCNM initiated the Asian Board of Nuclear Medicine (ABNM) in 2011, and is planning to have the first examination of ABNM in 2014. ABNM quality recognition is optional and does not interfere with the national specialty board of nuclear medicine. Candidates may enroll for and take a written exam each year at the Annual General Meeting of ARCCNM. ABNM and EBNM are working together to make the first exam successful.

Science and technology develop and change at a very fast pace. Nuclear medicine was born by hybridization of various fields of science and technology. Hybrid imaging became a hot topic again by the wide acceptance of PET/CT. PET/MRI has been receiving more and more interest from medical and business sides. Requirement of efficient training on anatomical imaging for nuclear medicine residents is growing. Close collaboration of nuclear medicine and radiology for better training and for better service to patients is needed. It is the responsibility of AOFNMB to make nuclear medicine trainees to be compatible with the global standards of training.

A disaster developed in Fukushima, Japan in March 2011, which raised a huge public concern regarding the harmful effect of radiation throughout the world. It adversely affected practice of nuclear medicine. Some patients canceled their nuclear medicine examinations because of fear of atomic energy. Providing sufficient information to the public is a responsibility of nuclear medicine specialists, which has not been sufficient enough in many countries. Japanese nuclear medicine specialists are providing information on the disaster of Fukushima nuclear power plant very actively through the JSNM website.[4] It has been the most popular website in Japan for a long time and helped to improve public awareness of nuclear medicine. Collaboration among the member states of AOFNMB for better public education is also a big challenge.

In this brief editorial, I summarized the history and perspectives of AOFNMB. Despite its half-century history, AOFNMB has a far way to go to achieve better communications and sharing of knowledge and experiences among the members, better education and training, and promotion of nuclear medicine practice in Asia and Oceania.
